# Patterns and Predictors of ADRD Among Racial and Ethnic Minority Groups

**DOI:** 10.1093/geroni/igaa057.2075

**Published:** 2020-12-16

**Authors:** Laura Zahodne, Cerise Elliott

## Abstract

This symposium addresses issues surrounding Alzheimer’s disease and related dementia (ADRD) across multiple racial/ethnic minority groups, including African Americans, Latinos, and Arab Americans. Using US national data, Kindratt and colleagues challenge the universality of the healthy migrant effect by comparing patterns of cognitive disability across US- and foreign-born Arab Americans. Arab Americans represent an increasingly visible ethnic minority group whose unique history has the potential to clarify knowledge about sociocultural influences on ADRD. Also using US national data, Garcia and colleagues examine within-group heterogeneity among Latinos. They conclude that the number of years and proportion of life spent with and without subjective cognitive impairment differ as a function of ancestry and nativity. Using data from two local communities, Diminich and colleagues investigate mechanisms underlying ADRD risk among Latinos by considering both stress responding and plasma-based AD biomarkers as predictors of Latino cognitive health. Lee and colleagues focus on social relationships and cognitive aging in a diverse, national cohort. They suggest that the quality of social support from social network members may uniquely affect the cognitive functioning of African Americans older adults. Finally, Cerise Elliott from the National Institute on Aging (NIA) will offer perspectives on how racial/ethnic minority group focused research can advance NIA’s goals related to understanding and eliminating ADRD inequalities. In total, this symposium highlights the need to disaggregate racial/ethnic groups, as well as the importance of incorporating both individual and contextual factors in order to fully understand patterns of ADRD risk and resilience.

